# Periprosthetic inflammation: from the cellular level to clinical implications

**DOI:** 10.1093/jbmrpl/ziaf154

**Published:** 2025-09-18

**Authors:** Jakub Maceška, Polina Navrátilová, Monika Pávková Goldbergová

**Affiliations:** Department of Pathophysiology, Faculty of Medicine, Masaryk University, 625 00 Brno, Czech Republic; Department of Pathophysiology, Faculty of Medicine, Masaryk University, 625 00 Brno, Czech Republic; Department of Pathophysiology, Faculty of Medicine, Masaryk University, 625 00 Brno, Czech Republic

**Keywords:** orthopedic implants, periprosthetic inflammation, inflammasome, cytokines, bone resorption

## Abstract

Periprosthetic inflammation is a crucial factor contributing to aseptic loosening, the leading cause of implant failures. Metallic debris, including nanoparticles, sub-micron particles, and ions, plays a central role in triggering inflammatory responses around orthopedic implants. Exposure to the debris activates macrophages via toll-like receptors and nucleotide-binding and oligomerization domain-like receptors, which in turn leads to the production of pro-inflammatory cytokines. This signaling cascade subsequently drives osteoclast activation, resulting in periprosthetic bone loss and, ultimately, implant loosening. Recent research has focused on strategies to prevent aseptic loosening by targeting the inflammation induced by metallic particles/ions. Pharmacological interventions aimed at modulating macrophage activation and inhibiting specific inflammatory pathways have shown promise in reducing osteoclast activity and excessive bone resorption. This review provides a comprehensive overview of the processes involved in the pathogenesis of periprosthetic inflammation, beginning with the release of metallic debris and its recognition by immune cells, followed by the inflammatory reactions that lead to osteoclastogenesis and bone loss. A detailed understanding of these molecular mechanisms is essential for the development of targeted approaches to prevent aseptic loosening, improve long-term patient outcomes, and alleviate the economic burden on healthcare systems.

## Graphical Abstract

**Graphical Abstract:**
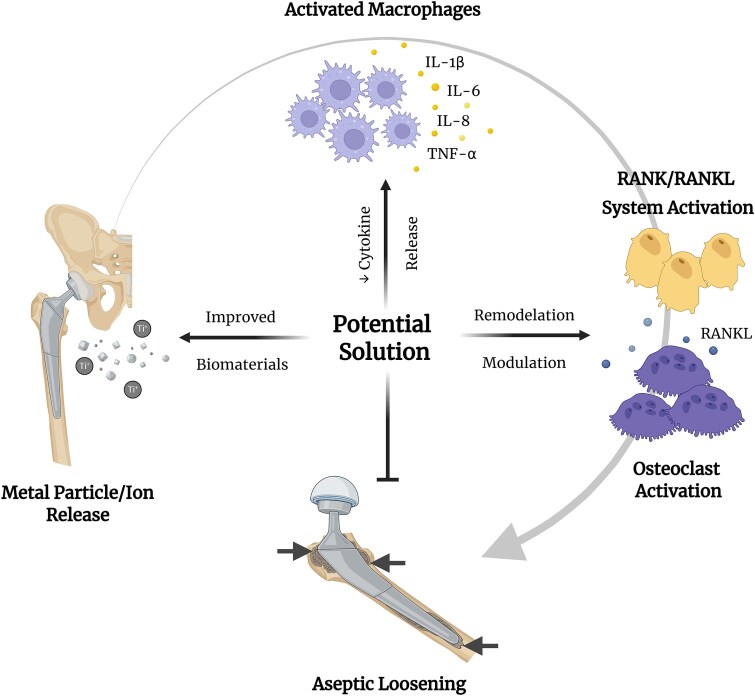

## Introduction

Orthopedic implants encompass a variety of devices designed for a range of applications. Total joint prostheses represent a major type of orthopedic implants, indicated for advanced stages of certain joint diseases, such as osteoarthritis.^1^ The number of total joint replacement surgeries is steadily growing, and this trend is expected to continue.^2^ Over the years, the design and materials used for prostheses have been improving to enhance biocompatibility and functionality. Due to bearing a significant weight load, the most commonly used materials in orthopedics are metals, such as stainless steels, cobalt-based alloys, titanium and titanium-based alloys, and, to a lesser extent, tantalum and zirconium alloys.^3^^,^^4^

Although materials currently used in orthopedics generally exhibit high endurance and low cytotoxicity, post-operative complications can still occur. One of the most prevalent complications is the development of local inflammation, mediated especially by macrophages. The pro-inflammatory milieu then stimulates osteoclasts, which subsequently resorb the periprosthetic bone tissue. In some cases, the bone loss can result in implant loosening, and revision surgery might be necessary. To prevent this, research efforts are focused on understanding and minimizing the causes of inflammatory reactions. Recent data show that revision surgery rates in total joint arthroplasties have decreased notably over the past decade from 15% to 9% in hip replacements and from 9% to 5% in knee replacements.^5^ This rapid decrease can be at least partially attributed to advanced materials and scientific progress in the field of implantology.

This review is focused on the topic of periprosthetic inflammation from a broad perspective, describing mechanisms leading to particle release, subsequent process of recognition by immune cells, and the formation of inflammasomes acting as key mediators of inflammation. Additionally, the connections between in vitro studies describing pro-inflammatory cytokine signaling in osteoclast differentiation and maturation, and clinical implications that focus on potential drug therapies, are shown. By making this complex overview, the article provides insights that deepen the understanding of periprosthetic inflammation and its impact on patients’ outcomes.

## Particle and ionic release mechanisms

**Figure 1:**
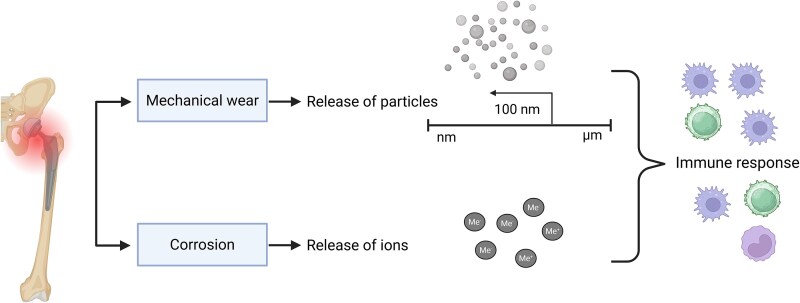
Mechanisms of particle and ionic release. Mechanical wear causes the release of particles ranging from nm to μm, with the majority of NPs. Corrosion causes the release of ions. Both particles and ions elicit an immune response. Me+/Me−, metal ions; NPs, nanoparticles. Created with BioRender.com.

The release of metal particles and ions from the implants is caused by a process called bio-tribocorrosion, a combination of mechanical and chemical degradation in specific tissue microenvironments (Figure 1).^6^ Mechanical wear occurs as a result of sliding of two surfaces over each other, leading to the release of particles from their superficial layers.^7^ Corrosion, on the other hand, refers to electrochemical reactions between metallic material and the surrounding environment.^8^ Based on different mechanisms, several subtypes of corrosion can be distinguished. This review examines the types of corrosion with the highest relevance to orthopedic implants, namely fretting corrosion, microabrasion-corrosion, pitting corrosion, crevice corrosion, and galvanic corrosion.

*Fretting corrosion* is caused by oscillatory micromovements between two metal components, a phenomenon commonly seen in joint prostheses. The corrosion rate depends on several factors including the type of material and the presence of fluid between the components of the implant.^9^

*Microabrasion-corrosion* refers to a degradation of the surface caused by abrasive particles. The particles can either: (1) adhere to a single surface and create linear grooves (known as two-body abrasion) or (2) roll between two surfaces resulting in indentations without a distinct pattern or direction (three-body abrasion). In both cases, the presence of abrasive particles disrupts the protective oxide layer (passivation film) on the metal surface, thereby increasing the corrosion rate.^10^^,^^11^

*Pitting corrosion* is characterized by a local degradation of metallic surfaces that extends into the metal, forming isolated cavities. This type of corrosion is caused by ions present in the biological microenvironment, which interact primarily with defective areas.^12^ A similar mechanism is involved in *crevice corrosion*, characterized by surface damage occurring under occluded areas, where an electrochemical cell is generated, consequently leading to the destabilization of the passive film.^12^^,^^13^

*Galvanic corrosion* occurs in the presence of two different metals immersed in a conductive fluid medium, such as blood, interstitial fluid, or saliva. The corrosion rate depends on many factors, including the specific types of metals, the characteristics of the fluid, and the areas of contact. Studies indicate that galvanic corrosion in orthopedic implants occurs at a minimal rate, with no considerable risk to surrounding tissues.^14^^,^^15^ However, this is not the case for implants in the oral environment, where galvanic corrosion is a major cause of complications associated with metallic devices, such as dental implants.^16^

Released particles from the implants can vary in size significantly, ranging from microparticles (MPs) to nanoparticles (NPs). According to the studies, the majority of the released particles are smaller than 100 nm.^17–21^ Predictions suggest that from metal-on-metal (MoM) prostheses, between 10^11^ and 10^14^ particles could be released annually,^18^^,^^19^ with wear rates estimated at approximately 1 mm^3^/yr from MoM implants.^22^ These data are related to well-functioning implants; however, malfunctioning implants can produce larger particles with significantly increased wear rates.^23^^,^^24^ In general, smaller particles are generated due to the repetitive movements between components of implants, while larger particles are released because of material fatigue.^25^ In contrast, the release of metal ions occurs as a result of different types of corrosion. The process of ion release can be accelerated by different environmental factors, such as extreme pH levels or the presence of biomolecules.^26^

## Immune recognition of implant degradation products and processes leading to cytokine release

Numerous studies have documented that released particles and ions significantly affect immune cells in various aspects, including the induction of inflammatory responses (see Tables S1-S3). Such responses can subsequently lead to bone degradation with severe impairment of implant osseointegration. Although evidence for these effects has existed for decades, the processes leading to cytokine release have only recently begun to be unraveled.

In general, the sensing of pathogen-associated molecular patterns (PAMPs) and damage-associated molecular patterns (DAMPs) by immune cells occurs through pattern recognition receptors (PRRs) exhibited by cells of the innate immune system.^27^ Various types of PRRs are described, including toll-like receptors (TLRs), retinoic acid-inducible gene I-like receptors, nucleotide-binding and oligomerization domain (NOD)-like receptors (NLRs), absent in melanoma 2-like receptors, C-type lectin receptors, and intracellular DNA and RNA sensors.^28^^,^^29^ The following paragraphs focus on the role of TLRs and NLRs, whose involvement has been documented in in vitro/in vivo models challenged with metal particles and ions potentially released from orthopedic implants. In a brief summary, the TLRs are known to upregulate the transcription of inflammatory cytokines, while certain NLRs are able to constitute components of inflammasomes, which convert certain inactive cytokines into their bioactive forms. However, recent studies indicate that these two signaling cascades influence one another, and the final release of matured cytokines occurs as a result of their interplay.^29^

### TLRs and TLR signaling

Toll-like receptors are represented by 10 types in humans (TLR1-TLR10) and are localized either extracellularly or in intracellular compartments.^28^ These receptors are able to recognize a large variety of PAMPs and are vital for the immune system’s ability to detect microorganisms.

The TLR signaling occurs through the recruitment of specific adaptor proteins, leading to the activation of transcription factors, such as NF-κB, interferon regulatory factors, cAMP response element-binding protein, and activating protein-1.^28^ Activation of these transcription factors leads to upregulation of the transcription of inflammatory cytokines, such as interleukin (IL)-1β, IL-6, IL-12, TNF-*α*, and interferons (IFNs),^30^^,^^31^ as well as sensor proteins constituting inflammasomes (particularly NLRP3, see “NLRs and their role in inflammasome formation”). Importantly, the cytokines are released in an inactive form and require cleavage for their activation. This process is mediated by converting enzymes, known as caspases. One of the most potent pro-inflammatory cytokines, IL-1β, requires such activation, which is governed by caspase-1 (formerly named “interleukin-1 converting enzyme”), an effector protein of inflammasomes. TNF-α, as well as some other cytokines, is activated through the cleavage governed by TNF-α converting enzyme (TACE or ADAM17), which can be activated by pro-inflammatory cytokines (eg, IL-1β) and transcription factors NF-κB and Elk-1.^32–34^

Several studies observed the TLRs involvement in response to implant debris. As for metals, Potnis et al.^35^ documented that the release of pro-inflammatory IL-8 in response to Co wear particles occurs through the activation of the TLR4 signaling pathway. They demonstrated the involvement of myeloid differentiation primary response 88 (MyD88) and IL-1 receptor associated kinase 1 proteins as well as the activation of the NF-κB pathway, all downstream effectors of the TLR4 pathway. Greenfield et al.^36^ showed that Ti particles with adherent bacterial debris activated both TLR2 and TLR4 pathways, whereas “endotoxin-free” particles activated neither TLR2 nor TLR4. Tao et al.^37^ demonstrated that CuO NPs can also activate the TLR4/MyD88/NF-κB pathway in J774A.1 macrophages. Similarly, Lawrence et al.^38^ reported the involvement of TLR4 in IL-6 release after stimulation with Co ions in MonoMac 6 cell line. Interestingly, Samelko et al.^39^ subjected THP-1 macrophages, primary human macrophages, and a murine in vivo model to cobalt alloy particles and showed only negligible involvement of TLR4 signaling, while inflammasome activation appeared to be “highly dominant” in mediating inflammatory reactions. Regarding the non-metalic components of implants, the activation of the TLR4 pathway by ultra-high-molecular-weight polyethylene (PE) debris was demonstrated by Hao et al.^40^ using primary human monocytes exposed to particles. Pearl et al.^41^ described similar effects on murine in vivo model and murine macrophages challenged with poly(methyl methacrylate) particles. In both studies, cells deficient in TLRs expressed significantly lower levels of pro-inflammatory cytokines than wild types.

### NLRs and their role in inflammasome formation

Nucleotide-binding and oligomerization domain-like receptors are a family of intracellular receptors responsible for mediating the initial innate immune response to PAMPs and DAMPs.^29^ In humans, there are 22 types of NLRs, which are divided into subfamilies based on their N-terminal effector domains: NOD-like receptor acidic transactivation domain (NLRA) 1), NOD-like receptor inhibitor of apoptosis domain (NLRB) 1), NOD-like receptor caspase activation and recruitment domain-containing (NLRC) (5), NOD-like receptor pyrin domain-containing (NLRP) (14), and NOD-like receptor X domain-containing (NLRX) 1).^42^ Among those, NLRP and NLRC subfamilies are the most studied due to their distinct roles in immune responses.^43^ Some NLRs serve as essential components of multiprotein complexes called inflammasomes,^44^ which are key elements involved in the mediation of inflammatory reactions, a fundamental mechanism of innate immune response.^45^

According to the classical concept, inflammasomes consist of sensors, adaptors, and effectors;^46^ in this model, the NLRs constitute a sensor. To date, the ability to form an inflammasome has been described in members of the NLRP and NLRC subfamilies (namely NLRP-1,3,6,7,9 and NLRC4) and some other proteins (absent in melanoma 2—AIM2, pyrin).^47^^,^^48^ If activated, the sensors recruit adaptors, typically apoptosis-associated speck-like protein, which subsequently recruit procaspase, followed by its conversion into the active form. Currently, two subtypes of inflammasomes have been described based on the effector caspase involved. The “canonical inflammasomes,” discovered in 2002 by Martinon et al.,^49^ work through the activation of procaspase-1. Active caspase-1 then cleaves and thereby activates pro-inflammatory cytokines pro-IL-1β, pro-IL-18, and pore-forming gasdermin D (GSDMD).^46^ The “non-canonical inflammasomes” activate caspase-4 or -5 in humans or caspase-11 in mice.^50^ Since the evidence concerning “non-canonical inflammasomes” is limited and research describing their association with implant debris is currently missing, we focus exclusively on the “canonical inflammasomes.”

**Figure 2:**
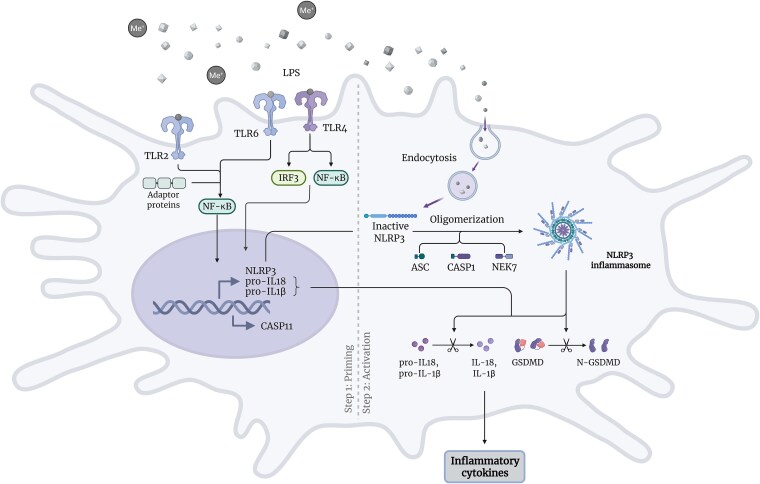
Activation of NLRP3 inflammasome. Metal debris binds to their respective PRRs (TLR2, TLR4, and TLR6) to activate NF-κB, which upregulates the gene transcription of NLRP3, CASP1, pro-IL-1β, and pro-IL-18. Inactive ubiquitinated NLRP3 undergoes oligomerization with ASC, NEK7, and CASP1, leading to inflammasome formation. Activated inflammasome cleaves pro-IL-1β, pro-IL-18, and GSDMD to active forms: IL-1β, IL-18, and the N-terminal of GSDMD. ASC, apoptosis-associated speck-like protein; CASP, caspase; GSDMD, gasdermin D; IRF, interferon regulatory factor; LPS, bacterial lipopolysaccharides; Me+, metal ions; NEK7, NIMA-related kinase 7; TLR, toll-like receptor. Created with BioRender.com.

The activation of “canonical inflammasomes” requires two sequential steps (Figure 2): priming and activation/assembly.^51^ Priming, as the first step, is essential for upregulating the transcription of NLRP3 and its substrates (ie, pro-IL-1β and pro-IL-18), and managing posttranslational modifications. Since the upregulation of NLRP3 expression takes about 2 h, rapid activation of NLRP3 is attributed to various posttranslational modifications, namely ubiquitination, phosphorylation, and others.^52^ The priming step is induced mainly by several downstream effector proteins of the TLR signaling pathway.^52^ Moreover, some evidence suggests the involvement of mitochondrial reactive oxygen species in triggering the priming process.^53^ The activation, as the second step, promotes the assembly of proteins (sensor–adaptor–effector) into a multimeric complex—the inflammasome. The interactions between these proteins lead to autocleavage and the formation of active caspase-1, which converts pro-IL-1β into the mature form IL-1β.^54^ Generally, NLRP3 can be activated by various seemingly unrelated stimuli, including pore-forming toxins, crystals, aggregates, and ATP.^46^ In the context of metal NPs, it was shown that ATP release plays a critical role in NLRP3 activation, along with subsequent ATP, ADP, and adenosine receptor signaling.^55^

To the best of our knowledge, the only currently known inflammasome involved in the reaction to metallic implant debris and ions is NLRP3. However, it is important to note that NLRP3 is, by far, the most extensively studied inflammasome, and only a limited number of studies have investigated the involvement of other types.

The activation of the NLRP3 inflammasome in response to metal particles with sizes ranging from nanometers to micrometers and with different surface modifications is supported by numerous studies (copper oxide NPs;^37^ iron oxide NPs;^56^ nano-TiO_2_;^55^ Ti, Cr, Mo MPs,^54^ and a review by Huang et al.^57^). The studies eliciting NLRP3 involvement employed either NLRP3 signaling inhibitors^37^^,^^54^^,^^55^ or used NLRP3 knockdown cells.^37^^,^^56^ In both experimental approaches, the NLRP3 inflammasome appeared to be pivotal for IL-1β secretion.

Apart from (nano)particles, inflammasome activation has also been observed after the addition of metal ions. In human alveolar bone-derived mesenchymal stromal cells cultured with Ti ions, a slight increase in NLRP3, caspase-1, and IL-1β was reported with the effect being further amplified after the addition of bacterial lipopolysaccharides (LPS). In this study, the activation of the AIM2 inflammasome in response solely to Ti ions was not confirmed.^58^ Similar results were obtained by Li et al.,^59^ where Ti ions activated NLRP3 in Jurkat T cells; however, the expression levels of other inflammasomes, particularly NLRP1, AIM2, and NLRC4, were not significantly affected.

## Metal particles and ions stimulate cytokine release in macrophages

### Cytokine release in vitro

The interplay between TLR and NLR signaling pathways results in the production of pro-inflammatory mediators. Numerous studies have investigated cytokine production induced by metal particles and/or ions. However, the experimental conditions in the conducted studies varied significantly, including the variability in cell types used in the study, exposure time, concentrations of particles/ions, stimulation with LPS, and characteristics of the used reagents (particles or ions). To unify and compare these results, we focus mainly on studies that investigated in vitro cytokine production by macrophages/monocytes stimulated with metal particles and ions (Tables S1-S3). In general, prolonged exposure to particles/ions caused more pronounced effects on cytokine levels.^60–63^ The pre-treatment with LPS markedly increased the overall cytokine levels;^62^^,^^64^^,^^65^ however, synergic effect between particles and LPS was observed only in some experimental designs (LPS and TiO_2_ NPs,^62^ LPS and Ti ions^65^). To emphasize the distinctions between different types of degradation products, we focus separately on: (1) particles of micron and submicron sizes, (2) NPs, and (3) ions.

According to the reviewed studies (Table S1), metal particles of micron and submicron sizes are potent stimulators of pro-inflammatory cytokines in macrophages. Almost all studies consistently reported the elevation of IL-1β,^35^^,^^60^^,^^64^^,^^66–69^ and some reported an increase in TNF-α,^60^^,^^64^^,^^67–72^ IL-6,^67–69^^,^^71^^,^^72^ and IL-8.^35^^,^^60^^,^^71^ Studies by Eger et al.^68^ on TiAlV particles and Kaufman et al.^71^ on TiAlV, CoCr, and Alumina particles tested an extended spectrum of cytokines and showed increased levels of IL-1α, IL-10, IFN-γ, granulocyte colony stimulating factor (G-CSF), granulocyte-macrophage colony-stimulating factor (GM-CSF), monocyte chemoattractant protein 1 (MCP-1), macrophage inflammatory protein α (MIPα), and others. However, the broader evidence is currently missing and both the cause (primary induction by particles vs secondary induction by already released cytokines) and the pathophysiological consequences on the human body remain unclear.

To examine whether smaller particles cause different effects, we summarized the literature focusing on the effects of NPs (Table S2), which constitute the majority of released particles from orthopedic implants (see “Particle and ionic release mechanisms”). Unfortunately, the literature focusing on NPs is relatively scarce and the experimental designs are not standardized. For instance, Nyga et al.^61^ reported significantly increased levels of IL-1β and TNF-α following 24 h exposure to Co NPs. Zhang et al.^62^ reported increased levels of IL-1β and TNF-α following 24 h exposure to TiO2 NPs, but showed that the same exposure to Ta NPs did not increase the levels of either cytokine. In this study, LPS significantly increased levels of both cytokines, but a synergic effect was observed only in TiO_2_ NPs. Pettersson et al.^73^ reported no increase in IL-1β levels following 18 h exposure to Ti NPs and TiO_2_ NPs. VanOs^74^ found that 60 and 700 nm Cr_2_O_3_ particles did not exert any stimulatory effect on TNF-α, MCP-1, and MIP-1α after 24 h exposure. Finally, Yang et al.^75^ analyzed the effect of 4-d exposure to Ti NPs on macrophages and showed significantly increased levels of IL-6 and TNF-α, while IL-4 and IL-10 were not significantly altered.

Similar to particles, metal ions also stimulated the release of pro-inflammatory cytokines by macrophages. Regardless of the material, metal ions caused an elevation of IL-1β^64–67^^,^^73^^,^^76^ and TNF-α^61^^,^^64^^,^^67^^,^^76^^,^^77^^,77^ production, as reported by the majority of the reviewed studies (Table S3). On the other hand, the results related to IL-6 are inconsistent. While some studies observed an elevation of IL-6 levels,^67^^,^^76^ others observed no change in its secretion.^65^^,^^67^^,^^76^ A more precise interpretation is complicated by the fact that higher concentrations of ions were toxic to cells, and a decrease in cell count was followed by a rapid decrease in cytokine levels.^66^^,^^67^ Pre-treatment with LPS had mostly stimulatory effect on cytokine levels.^65^

### Limitations of in vitro studies and cytokine release in vivo

It is important to emphasize that the degree of stimulatory effects of metal particles and ions on cytokine secretion in vitro does not fully reflect processes in periprosthetic patients’ tissues. The limitations of in vitro studies include the following:

in vitro models only partially replicate the complex in vivo environment with the presence of different cells and extracellular matrix;
the experiments mostly examined only one type of metallic pollutant, while a range of wear debris is released in vivo, ranging from nm to μm;
most of the in vitro studies examined the effect of 24 h stimulation, with more pronounced effects after 48 h stimulation documented by some studies,^60^^,^^63^ while long-term effects are unknown; and
the concentration of metal wear particles varied significantly in samples taken from periprosthetic patients’ soft tissues with up to 1000-fold differences between patients (Agins et al.^78^: 56-3700 μg of Ti per gram of dry tissue with an average of ~1 mg/g; Lohmann et al.^79^: 1.4-4604 μg of Co, Cr, and Ni/g). Nevertheless, the presence of metallic particles in periprosthetic tissues was reported in all patients after total hip arthroplasty (THA) by Willert et al.^80^ and Huber et al.,^81^ with grades of particle infiltration ranging from “few” (a few particles phagocytized in some spots and/or accumulated perivascularly) to “excessive” (tissue is overstuffed with particles). Similarly, concentrations of ions in tissues adjacent to the failed total knee prostheses had a large variability (Kurtz et al.^82^: in minimally damaged implants, median concentrations of Co, Cr, and Mo ions were 0.111, 1.80, and 0.179 μg/mL, respectively, while in severely damaged implants, median concentrations were 7.81, 5.26, and 0.713 μg/mL, respectively).

Considering these limitations, it is complicated to compare the degree of stimulatory effects of metal particles and ions on cytokine secretion in vitro and effects induced by metal pollution in vivo. In fact, large differences were observed even between animal studies and patients. In two studies on mice with Ti particle-induced calvaria osteolysis, Shao et al.^83^ exposed mice to 20 mg of Ti particles embedded on the calvarial bone surface and reported significant increase (compared to non-treated animals) in the expression of IL-1β (~175 pg/mL) and TNF-α (~215 pg/mL) in mouse calvariae, while Yu et al.^84^ exposed mice to 40 mg of Ti particles and reported similarly increased levels of IL-1β (~150 pg/mL) and TNF-α (~300 pg/mL). In contrast, Christiansen et al.^85^ assessed cytokine levels in patients with aseptic loosening of THA and reported significant increase of inflammatory cytokines in the peri-implant tissue (compared to the control group of patients receiving primary THA), in particular IL-1β (~8 pg/mL) and TNF-α (~10 pg/mL).

In conclusion, evidence from in vitro, in vivo, and patient studies consistently demonstrates that both metal particles and ions significantly stimulate macrophages to produce inflammatory mediators. Furthermore, metal ions elicit a stronger pro-inflammatory response compared to particulate debris,^64^^,^^86^ with Co, Mo, and Ni ions stimulating cytokine release even at low concentrations.^64^^,^^73^ The differences between different metal materials remain unclear and comparative studies on a complete spectrum of particulate debris and ions are needed to elucidate the “safest” material.

## Pro-inflammatory cytokines modulate bone remodeling by stimulation of osteoclasts

The immune and skeletal systems interact through shared regulatory molecules, primarily involving the RANK/RANKL/osteoprotegerin (OPG) pathway. The osteoclastogenic cytokine RANKL, produced by osteoblasts and osteocytes, binds to the RANK receptor on osteoclasts, driving their differentiation and bone-resorbing activity. Activated osteoclasts adhere to the bone and release hydrochloric acid and enzymes, thereby degrading the bone mineral and the organic matrix. In contrast, OPG, produced by osteoblasts and other mesenchymal-derived cells, binds to RANKL to inhibit osteoclast activation. The balance between RANKL and OPG regulates bone formation and resorption, maintaining bone homeostasis through a negative feedback loop.^87^ Beyond bone remodeling, the RANKL–RANK pathway also modulates immune responses by supporting dendritic cell survival,^88–90^ T cell/dendritic cell interactions,^88–91^ and lymph node development.^89^^,^^90^

**Figure 3:**
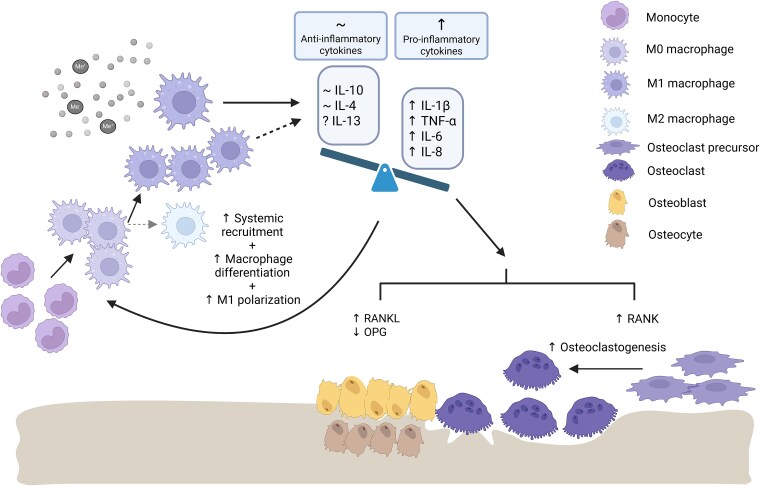
Effects of inflammatory cytokines on bone resorption. Metal debris induces the release of pro-inflammatory cytokines from macrophages. The imbalance in inflammatory mediators subsequently (1) increases recruitment of monocytes, promotes differentiation to macrophages, and polarization into M1 phenotype, thereby further increasing cytokine levels, and (2) promotes osteoclastogenesis by increasing production of RANKL and suppressing production of OPG by osteoblasts and osteocytes, and promoting the expression of RANK in osteoclast precursors. Me+/Me−, Metal ions; OPG, osteoprotegerin. Created with BioRender.com.

The imbalance between pro-inflammatory and anti-inflammatory signals propagates into an imbalance in the RANK/RANKL/OPG pathway (Figure 3). Certain pro-inflammatory cytokines upregulate RANKL production in osteoblasts and promote the expression of RANK in osteoclast precursors.^92^ The osteoclastogenic effect was observed in IL-1, IL-6, IL-8, IL-11, IL-15, IL-17, IL-32, and TNF-α, while IL-4, IL-10, IL-13, IL-18, IFN-γ, and IFN-β are considered anti-osteoclastogenic.^93^ Other cytokines, such as IL-7, IL-12, and IL-23, possess dual roles, depending on the specific (patho)physiological conditions.^93^ However, the situation is even more complicated by the fact that cytokines often influence each other, either synergically or antagonistically.^93^ In fact, the complex effect on bone resorption is shaped by both innate and adaptive immune responses, involving a complex network of signaling interactions among immune and bone cells.

## Particle-induced macroscopic changes in periprosthetic tissues and bone

As already mentioned, a variety of pro-inflammatory cytokines released from macrophages into the peri-implant tissue promote differentiation and activation of osteoclasts, ultimately resulting in bone degradation. This well-established mechanism observed in vitro is documented by in vivo models and patient studies. In animal models, metallic and PE particles induce large-scale changes in bone structure with deteriorative effects on implant stability and function (Table 1). Wear particles induced a significant decrease in implant fixation strength,^94–99^ impaired histomorphometric parameters of bone microarchitecture,^83^^,^^84^^,^^94–98^^,^^100–111^ and promoted a shift toward pro-osteoclastogenic signaling.^83^^,^^84^^,^^94^^,^^95^^,^^99^^,^^100^^,^^104^^,^^105^^,^^110^^,^^112–115^ Finally, the number of osteoclasts was elevated,^83^^,^^84^^,^^95^^,^^99–105^^,^^107^^,^^109–112^^,^^114–117^ along with an increase in the expression of osteoclast-associated proteins.^83^^,^^84^^,^^95^^,^^99–105^^,^^107^^,^^109^^,^^110^^,^^112^^,^^114^^,^^115^^,^^117^^,^^118^

**Table 1:** Effects of metal and PE particles on bone parameters in animal models.

	**Particles**	**Bone parameter**	**Reference**
**Histomorphometric parameters**	Ti, CoCrMo	↓ BMD	^83^ ^,^ ^84^^,^ ^95^^,^ ^98^^,^ ^100-105^
	Ti	↓ Bone volume (BV)	^105^ ^,^ ^106^
	Ti, CoCrMo, UHMWPE	↓ Bone volume percentage (BV/TV)	^83^ ^,^ ^84^^,^ ^94-98^^,^ ^100-111^
	Ti	↑ Bone surface/bone volume ratio (BS/BV)	^94^ ^,^ ^95^^,^ ^106^
	Ti	↑ Structure model index (SMI)	^94^ ^,^ ^95^
	Ti	↓ Bone area/total area (B.Ar/T.Ar)	^95^
	Ti	↓ Trabecular thickness (Tb.Th)	^95^ ^,^ ^106^
	Ti, UHMWPE	↓ Trabecular number (Tb.N)	^94-97^ ^,^ ^106^^,^ ^111^
	Ti	↑ Trabecular pattern factor (Tb.Pf)	^94^
**Mechanical parameters**	Ti	↓ Implant fixation strength	^94-99^
**Molecules involved in pro-/anti-osteoclastogenic signaling**	Ti, CoCrMo	↑ Inflammatory cytokines	^83^ ^,^ ^84^^,^ ^94^^,^ ^99^^,^ ^100^^,^ ^104^^,^ ^105^^,^ ^110^^,^ ^112-115^
	Ti	↑ RANKL	^83^ ^,^ ^95^^,^ ^105^^,^ ^110^
	Ti	↓ OPG	^83^ ^,^ ^94^^,^ ^105^
**Osteoclast-related parameters**	Ti, CoCrMo, UHMWPE, PMMA	↑ Number of osteoclasts	^83^ ^,^ ^84^^,^ ^95^^,^ ^99-105^^,^ ^107^^,^ ^109-112^^,^ ^114-117^
	Ti, CoCrMo, UHMWPE, PMMA	↑ Tartrate-resistant acid phosphatase (TRAP)	^83^ ^,^ ^84^^,^ ^99-105^^,^ ^107^^,^ ^109^^,^ ^110^^,^ ^112^^,^ ^114^^,^ ^115^^,^ ^117^^,^ ^118^
	Ti	↑ Serum cross-linked C-telopeptide of type I collagen (CTX-1)	^95^ ^,^ ^110^
	Ti	↑ Calcitonin receptor	^83^
	Ti	↑ Osteoclast-associated receptor (OSCAR)	^110^
	Ti, PMMA	↑ Cathepsin K	^83^ ^,^ ^84^^,^ ^117^
	Ti	↑ Nuclear factor of activated T cells c1 (NFATc1)	^83^ ^,^ ^84^

↑ Stimulatory effect.

↓ Inhibitory effect.

The studies analyzing samples of periprosthetic tissues of patients with total joint prostheses, taken either during revisions or postmortem, consistently reported areas of osteolytic cavities with sizes up to several milimeters.^81^^,^^119^^,^^120^ These cavities were filled with monocytes/macrophages, lymphocytes, necrotic cellular debris, and wear particles distributed both intra- and extra-cellularly.^81^ A varying number of macrophages was observed, including both mature and recently invaded macrophages.^80^ In some cases, foreign-body giant cells were also present.^79^^,^^80^ Lymphocytic infiltration consisted of both B-cells and T-cells, with T-cells being more prevalent and, in some patients, contributing to a type IV hypersensitivity reaction.^80^ Cytokine analysis of the peri-implant tissue of patients with aseptic loosening revealed significantly elevated levels of pro-osteoclastogenic IL-1β, IL-2, IL-6, IL-8, GM-CSF, and TNF-α, but also anti-osteoclastogenic IL-4, IL-10, and IFN-γ.^85^ Notably, the cytokine elevations were not reflected in systemic blood circulation.^85^

Despite the aforementioned deteriorative effect of inflammation on bone integrity, it is important to note that regulated and short-term inflammation plays an essential role in tissue regeneration. Recent findings indicate that inflammation is a highly regulated process, which is terminated by various mediators, such as resolvins, lipoxins, protectins, and maresins.^121^ These mediators reduce the recruitment of leukocytes, promote phagocytosis of apoptotic cells, and manage cytokine levels, thereby regulating the inflammatory response and contributing to wound healing and tissue regeneration.^121^ If not terminated, inflammation progresses to a chronic state and this sophisticated evolutionary mechanism becomes destructive for the body through the above-described mechanism.

## Strategies for preventing implant failure

Periprosthetic osteolysis can lead to implant loosening, a condition that is indicated for revision surgery.^122^ However, revision surgery multiplies risks for the patient, creates a significant financial burden on healthcare systems, and already-present bone loss might impair the osseointegration of a newly implanted device. To enhance the success rate of implantations, research is focused on developing wear-resistant and/or biocompatible materials and finding potential drug therapies.

### Material strategies for minimizing particle release

To prevent the complex immune reaction to metal debris with its detrimental consequences for implant longevity, modern implant designs have increasingly focused on minimizing metal particle release. This has led to the development and increased use of alternative bearing surfaces in total joint arthroplasty, with evidence of enhanced implant longevity. A meta-analysis by Yin et al.^123^ showed that MoM implants and metal-on-conventional PE have higher revision rates compared to other bearing surfaces, particularly to ceramic-on-ceramic (CoC), ceramic-on-conventional PE, ceramic-on-highly-crosslinked PE, and metal-on-highly-crosslinked PE. A more recent study by Higuchi et al.^124^ reported a significantly higher rate of osteolysis on radiographic imaging in the MoM group compared to CoC group (14.3% vs 2.1%) but no significant difference in failure rate between the two groups after 8 yr of follow-up. The authors note that the survivorship may differ with longer-term follow-up. However, in addition to implant longevity, mechanical properties must also be considered, as CoC implants have been associated with a risk of ceramic fracture and audible squeaking.^124–126^

### Pharmacological therapies

Numerous studies document various effective pharmacological strategies for the prevention of implant failure, tested in vitro, in animals, and used in patients. However, currently in 2025, no approved drug exists for the prevention of periprosthetic bone loss and subsequent implant failure. Drugs for preventing periprosthetic osteolysis can be potentially targeted at different steps of the underlying pathological process.

The first step is the development of local inflammation driven by various cytokines predominantly produced by immune cells in response to implant debris. Therefore, blocking of these cytokines appears as a logical approach for preventing development of aseptic loosening. Etanercept, a TNF-α inhibitor, effectively abrogated particle induced osteolysis in mouse models.^112^^,^^118^ The administration of anti-osteoclastogenic IL-4 prevented particle-induced osteolysis in mouse models^107^ and IL-10 gene therapy abrogated osteoclastogenic effect of Ti particles, both in vitro and in vivo.^115^ Statins have been shown to counteract the induction of IL-6^127^ and IL-8^128^ in vitro. In patients, use of statins has been associated with reduced risk of developing femoral osteolysis following THA.^129^ Cyclooxygenase 2 inhibitors, such as celecoxib, decreased the production of pro-inflammatory prostaglandin E2, and the beneficial effect on osteolysis prevention has been shown in animal models.^114^^,^^130^ Interestingly, a macrolide antibiotic erythromycin in the coating of Ti pins mitigated particle-induced bone loss in rats^108^ and oral erythromycin reduced the aseptic inflammation in patients after THA.^131^ Inhibition of NF-κB by pyrrolidine dithiocarbamate,^113^ celastrol,^103^ and icariin^83^^,^^100^ subsequently reduced the expression of inflammatory cytokines, therefore mitigating particle-induced bone resorption in animal models. Reduction in particle-induced inflammation was observed in vitro, and/or in vivo after using kaempferol,^84^ proteasome inhibitor bortezomib,^63^ and *SOST* gene suppression.^105^

The second step in the pathogenesis of aseptic loosening is the activation of osteoclasts caused by an imbalance in the regulatory RANK/RANKL/OPG pathway. Inhibitors of pro-osteoclastogenic RANKL are approved for the treatment of osteoporosis^132^ and denosumab, as the most well-known RANKL inhibitor, has been shown to effectively prevent periprosthetic bone loss in patients after arthroplasties.^133^ In two meta-analyses, denosumab was superior to other drugs tested for mitigating periprosthetic bone loss in patients after THAs.^134^^,^^135^ Bisphosphonates are another group of drugs that reduce osteoclast activity by, for example, promoting osteoclast apoptosis and decreasing osteoclast progenitor development.^136^ Their use has been effective in animal models of particle-induced osteolysis^116^^,^^137^ and in patient studies appeared as one of the most promising approach for prevention of implant failure.^134^^,^^135^^,^^138^ Similarly, an anti-osteoporotic drug strontium ranelate reduced the degree of particle-mediated osteolysis in mice by simultaneously promoting osteoblast differentiation and suppressing osteoclastogenesis.^94^^,^^106^ Raloxifene, a selective estrogen receptor modulator, has significantly enhanced prosthetic function and overall quality of life of postmenopausal women after THAs due to its anti-osteoclastogenic effect.^139^ The anti-osteoclastogenic effect was also observed in animal models of particle-induced osteolysis with the use of sirtuin 3 inhibitor (3-(1H-1,2,3-triazol-4-yl) pyridine—3-TYP),^104^ biochanin A,^110^ acetyl-11-keto-β-boswellic acid (AKBA),^102^ formononetin,^101^ glycyrrhizin,^117^ irisin,^140^ and the histone deacetylase inhibitor quisinostat.^109^

Another approach to mitigating wear debris-induced bone loss involves supporting bone formation. This approach has been tested on intermittently administered PTH (iPTH), which promotes bone formation by stimulating osteoblasts.^141^ In animal studies, iPTH monotherapy^95–98^ and combination therapy with zoledronic acid (bisphosphonate)^95^^,^^96^ mitigated particle-induced osteolysis. The combination therapy was more effective.^95^^,^^96^

## Conclusion

The use of metal materials in various medical fields, especially orthopedics, is inevitably linked to particle and ion release. Macrophages, among other cells, sense the released debris and trigger a complex response that leads to the activation of TLR and NLR signaling pathways, contributing to the production of pro-inflammatory cytokines. The reviewed studies highlight that metal particles of micron and submicron sizes, NPs, and metal ions, significantly stimulate macrophages to produce key inflammatory cytokines, such as IL-1β, TNF-α, and IL-6. However, inconsistencies in the literature regarding cytokine responses, particularly the role of metal material and the size of particles, indicate a need for further research to clarify these effects and their underlying mechanisms.

The imbalance in immune response induced by metal particles and ions significantly impacts bone remodeling, mostly through the promotion of osteoclast differentiation and activation. The disrupted balance between bone formation and degradation may result in periprosthetic osteolysis and subsequent aseptic loosening. This was traditionally indicated for revision surgery as the only treatment option. However, novel implant designs and non-invasive treatment strategies now offer effective alternatives for preventing or treating extensive periprosthetic bone degradation.

Overall, this review underscores the importance of standardizing experimental conditions in future studies to better characterize the biological effects of metal degradation products. A deeper understanding of their inflammatory potential and impact on bone remodeling will be essential to improving the long-term stability of orthopedic implants and minimizing adverse outcomes for patients.

## Supplementary Material

Supplementary_materials_table_1_ziaf154

Supplementary_materials_table_2_ziaf154

Supplementary_materials_table_3_ziaf154

## Data Availability

All data presented in this article are available from the author upon request.
