# Supplementary material for: Periprosthetic inflammation: from the cellular level to clinical implications
Source: JBMR Plus. 2025 Sep 18;9(11):ziaf154. doi: 10.1093/jbmrpl/ziaf154 (PMC12526913; doi:10.1093/jbmrpl/ziaf154)
Supplement: Supplementary_materials_table_1_ziaf154 [file supplementary_materials_table_1_ziaf154.pdf]

Supplementary table I: effects of metal particles on cytokine release in macrophages

Study	Particle characterization	Cells	Exposure time	Significant increase (↑) in cytokine levels	No/not-significant increase (→) in cytokine levels; decrease (↓) in cytokine levels
**Co alloy particles (mainly Co-Cr-Mo alloy)**					
Caicedo et al.[67]	Mean diameter: 2 µm; size range: 1–10 µm	THP-1 human monocytic cell line	24 h	↑ IL-1β at 50:1 particles:cells	→ IL-1β at 1:1, 5:1, 10:1 particles:cells
				↑ TNF-α at 50:1 particles:cells	→ TNF-α at 1:1, 5:1, 10:1 particles:cells
		Human primary monocytes / macrophages	48 h	↑ IL-1β at 10:1 particles:cells	
				↑ IL-6 at 10:1 particles:cells	
				↑ TNF-α at 10:1 particles:cells	
Caicedo et al.[66]	Mean diameter: 2 µm; size range: 1–10 µm	THP-1 human monocytic cell line	24 h	↑ IL-1β at 10:1 particles:cells	→ IL-1β at 5:1, 20:1 particles:cells
		Human primary monocytes/macrophages		↑ IL-1β at 10:1 particles:cells	
Dalal et al.[60]	Mean diameter: 1.1 µm	THP-1 human monocytic cell line	24 h	↑ IL-1β at 50:1 particles:cells	→ IL-1β at 5:1, 10:1, 100:1 particles:cells
					→ IL-6 at 5:1, 10:1, 50:1, 100:1 particles:cells
				↑ IL-8 at 5:1, 10:1, 50:1, and 100:1	
				↑ TNF-α at 100:1 particles:cells	→ TNF-α at 5:1, 10:1, 50:1 particles:cells
			48 h	↑ IL-1β at 5:1 and 100:1 particles:cells	→ IL-1β at 10:1, 50:1 particles:cells
				↑ IL-8 at 5:1 and 10:1 particles:cells	→ IL-8 at 50:1, 100:1 particles:cells
Potnis et al.[35]	Mean diameter: 0.81 µm; size range: 1–10 µm	THP-1 human monocytic cell line	24 h	↑ IL-1β at 1000:1 particles:cells	→ IL-1β at 10:1, 100:1, 200:1, 500:1
				↑ IL-8 at 500:1 particles:cells	→ IL-8 at 10:1, 100:1, 200:1, 1000:1 particles:cells
					→ TNF-α at 10:1, 100:1, 200:1, 500:1, 1000:1
Yadav et al.[64]	Mean diameter: 0.9 µm	Human primary monocytes/macrophages	24 h	↑ IL-1β at 10:1 particles:cells	
				↑ TNF-α at 10:1 particles:cells	
Kaufman et al.[71]	Mean diameter: 0.48 µm	Human primary monocytes/macrophages	24 h		→ IL-1α at 853:1 particles:cells
					→ IL-1β at 853:1 particles:cells
				↑ IL-6 at 853:1 particles:cells	
				↑ IL-8 at 853:1 particles:cells	
					→ IL-10 at 853:1 particles:cells
				↑ TNF-α at 853:1 particles:cells	
					→ MCP-1 at 853:1 particles:cells
					→ GM-CSF at 853:1 particles:cells
Lacey et al.[72]	Size range: 0.5–3 µm	Murine primary bone marrow-derived macrophages	24 h		→ IL-1β at 10^7 particles/mL
					→ IL-6 at 10^7 particles/mL
					→ TNF-α at 10^7 particles/mL
					→ IFN-γ at 10^7 particles/mL
**Ti-Al-V alloy particles**					
Dalal et al.[60]	Mean diameter: 1.3 µm	THP-1 human monocytic cell line	24 h	↑ IL-1β at 100:1 particles:cells	→ IL-1β at 5:1, 10:1, 50:1 particles:cells
					→ IL-6 at 5:1, 10:1, 50:1, 100:1 particles:cells
					→ IL-8 at 5:1, 10:1, 50:1, 100:1 particles:cells
					→ TNF-α at 5:1, 10:1, 50:1, 100:1 particles:cells
			48 h	↑ IL-1β at 10:1, 50:1, 100:1 particles:cells	→ IL-1β at 5:1 particles:cells
				↑ IL-8 at 10:1, 100:1 particles:cells	→ IL-8 at 5:1,50:1 particles:cells
Yadav et al.[64]	Mean diameter: 1.2 µm	Human primary monocytes/macrophages	24 h	↑ IL-1β at 10:1 particles:cells	
				↑ TNF-α at 10:1 particles:cells	
Eger et al.[68]	NOT SPECIFIED	Murine primary bone marrow-derived macrophages	24 h	↑ IL-1α at 1293 particles/mm^2	
				↑ IL-1β at 1293 particles/mm^2	
				↑ IL-2 at 1293 particles/mm^2	
				↑ IL-6 at 1293 particles/mm^2	
				↑ IL-10 at 1293 particles/mm^2	
				↑ TNF-α at 1293 particles/mm^2	
				↑ IFN-γ at 1293 particles/mm^2	
				↑ G-CSF at 1293 particles/mm^2	
				↑ MCP-1 at 1293 particles/mm^2	
Kaufman et al.[71]	Mean diameter: 0.74 µm	Human primary monocytes/macrophages	24 h		→ IL-1α at 370:1 particles:cells
				↑ IL-1β at 370:1 particles:cells	
				↑ IL-6 at 370:1 particles:cells	
				↑ IL-8 at 370:1 particles:cells	
				↑ IL-10 at 370:1 particles:cells	
				↑ TNF-α at 370:1 particles:cells	
				↑ MCP-1 at 370:1 particles:cells	
				↑ GM-CSF at 370:1 particles:cells	
Lacey et al.[72]	Size range: 0.5–3 µm	Murine primary bone marrow-derived macrophages	24 h		→ IL-1β at 10^7 particles/mL
				↑ IL-6 at 10^7 particles/mL particles:cells	
				↑ TNF-α at 10^7 particles/mL particles:cells	
					→ IFN-γ at 10^7 particles/mL
**TiO_2 particles**					
Vallés et al.[69]	Mean diameter: 0.45 µm; size range: 0.1–1.5 µm	THP-1 human monocytic cell line	24 h	↑ IL-1β at 0.5, 5, 50 ng/cell	
				↑ IL-6 at 0.5, 5, 50 ng/cell	
				↑ TNF-α at 0.5, 5, 50 ng/cell	
		Human primary monocytes/macrophages		↑ IL-1β at 50 ng/cell	→ IL-1β at 0.5, 5 ng/cell
				↑ IL-6 at 0.5, 5, 50 ng/cell	
				↑ TNF-α at 0.5, 5, 50 ng/cell	
**Ti particles**					
Vallés et al.[69]	Mean diameter: 3.32 µm; size range: 1–15 µm	THP-1 human monocytic cell line	24 h	↑ IL-1β at 0.5, 5, 50 ng/cell	
				↑ IL-6 at 0.5, 5, 50 ng/cell	
				↑ TNF-α at 0.5, 5, 50 ng/cell	
		Human primary monocytes/macrophages		↑ IL-1β at 50 ng/cell	→ IL-1β at 0.5, 5 ng/cell
				↑ IL-6 at 0.5, 5, 50 ng/cell	
				↑ TNF-α at 0.5, 5, 50 ng/cell	
Wang et al.[106]	Mean diameter: 2.92 µm	RAW264.7 murine macrophages	24 h	↑ IL-1β at 10:1, 50:1, 100:1 particles:cells	
				↑ TNF-α at 50:1, 100:1 particles:cells	→ TNF-α at 10:1 particles:cells
				↑ IL-6 50:1, 100:1 particles:cells	→ IL-6 at 10:1 particles:cells
Mao et al.[63]	84 % of the particles < 10 µm in diameter	RAW264.7 murine macrophages	24 h	↑ IL-1β at 0.1 mg/mL	
				↑ TNF-α at 0.1 mg/mL	
				↑ IL-6 at 0.1 mg/mL	
				↑ IL-10 at 0.1 mg/mL	
				↑ MCP-1 at 0.1 mg/mL	
				↑ MIP-1α at 0.1 mg/mL	

**ZrO_2 particles**					
Dalal et al.[60]	mean diameter: 0.5 µm	THP-1 human monocytic cell line	24 h	↑ IL-1β at 50:1, 100:1 particles:cells	→ IL-1β at 5:1, 10:1 particles:cells
					→ IL-6 at 5:1, 10:1, 50:1, 100:1 particles:cells
					→ IL-8 at 5:1, 10:1, 50:1, 100:1 particles:cells
					→ TNF-α at 5:1, 10:1, 50:1, 100:1 particles:cells
			48 h	↑ IL-1β at 50:1, 100:1 particles:cells	→ IL-1β at 5:1, 10:1 particles:cells
				↑ IL-8 at 100:1 particles:cells	→ IL-8 at 5:1, 10:1, 50:1 particles:cells
**Zr-alloy particles**					
Dalal et al.[60]	Mean diameter: 0.9 µm	THP-1 human monocytic cell line	24 h	↑ IL-1β at 100:1 particles:cells	→ IL-1β at 5:1, 10:1, 50:1 particles:cells
					→ IL-6 at 5:1, 10:1, 50:1, 100:1 particles:cells
					→ IL-8 at 5:1, 10:1, 50:1, 100:1 particles:cells
					→ TNF-α at 5:1, 10:1, 50:1, 100:1 particles:cells
			48 h	↑ IL-1β at 100:1 particles:cells	→ IL-1β at 5:1, 10:1, 50:1 particles:cells
				↑ IL-8 at 50:1, 100:1 particles:cells	→ IL-8 at 5:1, 10:1 particles:cells
**Alumina particles**					
Kaufman et al.[71]	Mean diameter: 0.23 µm	Human primary monocytes/macrophages	24 h		→ IL-1α at 3732:1 particles:cells
				↑ IL-1β at 3732:1 particles:cells	
					→ IL-6 at 3732:1 particles:cells
				↑ IL-8 at 3732:1 particles:cells	
					→ IL-10 at 3732:1 particles:cells
					→ TNF-α at 3732:1 particles:cells
				↑ MCP-1 at 3732:1 particles:cells	
					→ GM-CSF at 3732:1 particles:cells

↑ stimulatory effect on cytokine production
→ no effect on cytokine production
↓ inhibitory effect on cytokine production
Note: ↑/↓ counted when a study reported statistically significant increase/decrease of cytokine production; statistically insignificant change counted as →
