# Supplementary material for: Periprosthetic inflammation: from the cellular level to clinical implications
Source: JBMR Plus. 2025 Sep 18;9(11):ziaf154. doi: 10.1093/jbmrpl/ziaf154 (PMC12526913; doi:10.1093/jbmrpl/ziaf154)
Supplement: Supplementary_materials_table_2_ziaf154 [file supplementary_materials_table_2_ziaf154.pdf]

Supplementary table II: effects of metal nanoparticles on cytokine release in macrophages					
**Ti NPs**					
**Study**	**Particle characterization**	**Cells**	**Exposure time**	**Significant increase (↑) in cytokine levels**	**No/not-significant increase (→) in cytokine levels; decrease (↓) in cytokine levels**
Yang et al.[75]	Mean diameter: 52.59 nm	RAW264.7 murine macrophages	4 days		→ IL-4 at 0,1 mg/mL
				↑ IL-6 at 0,1 mg/mL	
					→ IL-10 at 0,1 mg/mL
				↑ TNF-α at 0,1 mg/mL	
**TiO_2 NPs**					
Zhang et al.[62]	Mean diameter: 54.3 nm	THP-1 human monocytic cell line	24 h	↑ IL-1β at 500 µg/mL	→ IL-1β at 100 µg/mL
				↑ TNF-α at 500 µg/mL	→ TNF-α at 100 µg/mL
**Ta NPs**					
Zhang et al.[62]	Mean diameter: 67.9 nm	THP-1 human monocytic cell line	24 h		→ IL-1β at 100, 500 µg/mL
					→ TNF-α at 100, 500 µg/mL
**Co NPs**					
Nyga et al.[61]	Mean diameter: 28 nm	U937 human monocytic cell line	24 h	↑ IL-1β at 10 µg/mL	
				↑ TNF-α at 10 µg/mL	
**Cr_2O_3 NPs**					
VanOs et al.[74]	Mean diameter: 60 nm and 700 nm	J774A.1 mouse macrophages	20–24 h		→ TNF-α at 100, 200, 300, 400 μm^3 per cell
					→ MCP-1 at 100, 200, 300, 400 μm^3 per cell
					→ MIP-1α at 100, 200, 300, 400 μm^3 per cell

↑ stimulatory effect on cytokine production

→ no effect on cytokine production

↓ inhibitory effect on cytokine production

Note: ↑/↓ counted when a study reported statistically significant increase/decrease of cytokine production; statistically insignificant change counted as →
