# Supplementary material for: Periprosthetic inflammation: from the cellular level to clinical implications
Source: JBMR Plus. 2025 Sep 18;9(11):ziaf154. doi: 10.1093/jbmrpl/ziaf154 (PMC12526913; doi:10.1093/jbmrpl/ziaf154)
Supplement: Supplementary_materials_table_3_ziaf154 [file supplementary_materials_table_3_ziaf154.pdf]

Supplementary table III: effects of metal ions on cytokine release in macrophages

Study	Cells	LPS treatment	Exposure time	Significant increase (↑) in cytokine levels	No/not-significant increase (→) in cytokine levels; decrease (↓) in cytokine levels
**Ti ions**					
Pettersson et al.[65]	Human primary monocytes/macrophages	-	24 h		→ IL-1α at 25 μM
					→ IL-1β at 25 μM
					→ IL-2 at 25 μM
					→ IL-4 at 25 μM
					→ IL-6 at 25 μM
					→ IL-8 at 25 μM
					→ IL-10 at 25 μM
					→ IL-12 at 25 μM
					→ IL-17α at 25 μM
					→ TNF-α at 25 μM
					→ IFN-γ at 25 μM
					→ GM-CSF at 25 μM
		100 ng/mL LPS			→ IL-1α at 25 μM
				↑ IL-1β at 25 μM	
					→ IL-2 at 25 μM
					→ IL-4 at 25 μM
					→ IL-6 at 25 μM
					→ IL-8 at 25 μM
				↑ IL-10 at 25 μM	
					→ IL-12 at 25 μM
					→ IL-17α at 25 μM
					→ TNF-α at 25 μM
				↑ IFN-γ at 25 μM	
					→ GM-CSF at 25 μM
	THP-1 human monocytic cell line	-			→ IL-1β at 25, 50, 100, 200, 400, 800 μM
		100 ng/mL LPS		↑ IL-1β at 50 μM, 100 μM, 200 μM, 400 μM	→ IL-1β at 25, 800 μM
Pettersson et al.[73]	THP-1 human monocytic cell line	100 ng/mL LPS	18 h	↑ IL-1β at 62, 125, 250, 500 μM	→ IL-1β at 31 μM
Wang et al.[76]	U937 human monocytic cell line	100 ng/mL LPS	24 h	↑ IL-1β at 1, 10, 100 ng/mL	→ IL-1β at 0.01, 0.1 ng/mL
				↑ IL-6 at 0.1, 1, 10, 100 ng/mL	→ IL-6 at 0,01 ng/mL
				↑ TNF-α at 0.01, 0.1, 1, 10, 100 ng/mL	
					→ TGF-β1 at 0.01, 0.1 ng/mL; ↓ TGF-β1 at 1, 10, 100 ng/mL
	Human primary monocytes/macrophages			↑ IL-1β at 1, 10, 100 ng/mL	→ IL-1β at 0.01, 0.1 ng/mL
				↑ IL-6 at 10, 100 ng/mL	→ IL-6 at 0.01, 0.1, 1 ng/mL
				↑ TNF-α at 100 ng/mL	→ TNF-α at 0.01, 0.1, 1, 10 ng/mL
					→ TGF-β1 at 0.01, 0.1 ng/mL; ↓ TGF-β1 at 1, 10, 100 ng/mL
**Cr ions**					
Caicedo et al.[67]	THP-1 human monocytic cell line	-	24 h	↑ IL-1β at 0.1, 0.2 mM	→ IL-1β at 0.01 mM
				↑ TNF-α at 0.1, 0.2 mM	→ TNF-α at 0.01 mM
	Human primary monocytes/macrophages		48 h		→ IL-1β at 0.1 mM
					→ IL-6 at 0.1 mM
					→ TNF-α at 0.1 mM
Caicedo et al.[66]	THP-1 human monocytic cell line	-	24 h	↑ IL-1β at 0.1, 0.2 mM	→ IL-1β at 0.01, 0.5 mM
	Human primary monocytes/macrophages			↑ IL-1β at 0.1 mM	
Pettersson et al.[65]	Human primary monocytes/macrophages	-	24 h		→ IL-1β at 5, 25 μM
		100 ng/mL LPS			→ IL-1β at 5, 25 μM
Wang et al.[76]	U937 human monocytic cell line	100 ng/mL LPS	24 h	↑ IL-1β at 100 ng/mL	→ IL-1β at 0.01, 0.1, 1, 10 ng/mL
					→ IL-6 at 0.01, 0.1, 1, 10, 100 ng/mL
				↑ TNF-α at 100 ng/mL	→ TNF-α at 0.01, 0.1, 1, 10 ng/mL
					→ TGF-β1 at 0.01, 0.1, 1, 10, 100 ng/mL
	Human primary monocytes/macrophages			↑ IL-1β at 100 ng/mL	→ IL-1β at 0.01, 0.1, 1, 10 ng/mL
					→ IL-6 at 0.01, 0.1, 1, 10, 100 ng/mL
				↑ TNF-α at 10, 100 ng/mL	→ TNF-α at 0.01, 0.1, 1 ng/mL
					→ TGF-β1 at 0.01, 0.1 ng/mL; ↓ TGF-β1 at 1, 10, 100 ng/mL
Catelas et al.[77]	J774 murine monocytic cell line	-	24 h	↑ TNF-α at 150, 250, 350 ppm	→ TNF-α at 50 ppm; ↓ TNF-α at 500 ppm
**Co ions**					
Caicedo et al.[67]	THP-1 human monocytic cell line	-	24 h	↑ IL-1β at 0.01, 0.1 mM	→ IL-1β at 0.2 mM
					→ TNF-α at 0.01, 0.1, 0.2 mM
	Human primary monocytes/macrophages		48 h	↑ IL-1β at 0.1 mM	
				↑ IL-6 at 0.1 mM	
				↑ TNF-α at 0.1 mM	
Caicedo et al.[66]	THP-1 human monocytic cell line	-	24 h	↑ IL-1β at 0.01, 0.1 mM	→ IL-1β at 0.2, 0.5 mM
	Human primary monocytes/macrophages			↑ IL-1β at 0.1 mM	
Pettersson et al.[65]	Human primary monocytes/macrophages	-	24 h		→ IL-1β at 5, 25 μM
		100 ng/mL LPS			→ IL-1β at 5, 25 μM
Wang et al.[76]	U937 human monocytic cell line	100 ng/mL LPS	24 h		→ IL-1β at 0.01, 0.1, 1, 10, 100 ng/mL
					→ IL-6 at 0.01, 0.1, 1, 10, 100 ng/mL
					→ TNF-α at 0.01, 0.1, 1, 10, 100 ng/mL
					→ TGF-β1 at 0.01, 0.1, 1, 10, 100 ng/mL
	Human primary monocytes/macrophages				→ IL-1β at 0.01, 0.1, 1, 10, 100 ng/mL
					→ IL-6 at 0.01, 0.1, 1, 10, 100 ng/mL
				↑ TNF-α at 10, 100 ng/mL	→ TNF-α at 0.01, 0.1, 1 ng/mL
					→ TGF-β1 at 0.01, 0.1, 1; ↓ TGF-β1 at 10, 100 ng/mL
Catelas et al.[77]	J774 murine monocytic cell line	-	24 h	↑ TNF-α at 4, 6, 8, 10 ppm	→ TNF-α at 2 ppm
**Mo ions**					
Caicedo et al.[67]	THP-1 human monocytic cell line	-	24 h	↑ IL-1β at 0.01, 0.1, 0.2 mM	
				↑ TNF-α at 0.01, 0.1, 0.2 mM	
	Human primary monocytes/macrophages		48 h	↑ IL-1β at 0.1 mM	
				↑ IL-6 at 0.1 mM	
					→ TNF-α at 0.1 mM

Caicedo et al.[66]	THP-1 human monocytic cell line	-	24 h	↑ IL-1β at 0.01, 0.1, 0.2 mM	→ IL-1β at 0.5 mM
	Human primary monocytes/macrophages			↑ IL-1β at 0.1 mM	
Pettersson et al.[65]	Human primary monocytes/macrophages	-	24 h		→ IL-1β at 5, 25 μM
		100 ng/mL LPS		↑ IL-1β at 25 μM	→ IL-1β at 5 μM
**Ni ions**					
Caicedo et al.[67]	THP-1 human monocytic cell line	-	24 h	↑ IL-1β at 0.01, 0.1 mM	→ IL-1β at 0.2 mM
				↑ TNF-α at 0.01, 0.1 mM	→ TNF-α at 0.2 mM
	Human primary monocytes/macrophages		48 h		→ IL-1β at 0.1 mM
					→ TNF-α at 0.1 mM
					→ IL-6 at 0.1 mM
Caicedo et al.[66]	THP-1 human monocytic cell line	-	24 h		→ IL-1β at 0.01, 0.1, 0.2, 0.5 mM
	Human primary monocytes/macrophages				→ IL-1β at 0.1 mM

↑ stimulatory effect on cytokine production
→ no effect on cytokine production
↓ inhibitory effect on cytokine production
Note: ↑/↓ counted when a study reported statistically significant increase/decrease of cytokine production; statistically insignificant change counted as →
